# Editorial: Microbial Dynamics During Industrial Rearing and Processing of Insects

**DOI:** 10.3389/fmicb.2021.775603

**Published:** 2021-11-02

**Authors:** Leen Van Campenhout, Jørgen Eilenberg

**Affiliations:** ^1^Research Group for Insect Production and Processing, Department of Microbial and Molecular Systems (M2S), Geel Campus, KU Leuven, Geel, Belgium; ^2^Leuven Food Science and Nutrition Research Centre (LFoRCe), KU Leuven, Leuven, Belgium; ^3^Department of Plant and Environmental Sciences, Faculty of Science, University of Copenhagen, Copenhagen, Denmark

**Keywords:** insect, production, rearing, processing, microbiome, dynamics, pathogen, interaction

Today, a number of insect species are farmed at a large scale. The major aim of mass production is insect biomass, which is then processed into food, animal feed, or pet food. The insect biomass can eventually be fragmented and purified to deliver biochemicals. Alternatively, the major aim of insect rearing can rather be waste conversion, with the resulting insect biomass as a useful byproduct improving the circularity of waste processing. Whatever the objective of the industrial cultivation and processing of insects, microbiological questions and challenges arise on many occasions in any insect production and processing project. Insects naturally harbor a rich microbiota, containing organisms that in some way contribute to, or conversely partially or fully obstruct the application. As industrial applications for insects make progress in versatility and scale, so does the research, and this is well-reflected in this Research Topic. The Topic covers microbiological questions and challenges related to the rearing, processing, and storage of insects, and it also addresses insect-related final products.

The major part of the papers relates to the rearing phase, and the share of papers envisaging processing and end products is smaller. Most of the research presented considers the dynamics of the whole microbiota in the insects (and the related environment), while only a few papers (additionally) focus on the role of specific microbial species.

The papers in the Topic related to microbial dynamics during the rearing phase all focus on black soldier fly larvae (BSFL), *Hermetia illucens*. The range of substrates BSFL can feed on is extensive, and a fascinating question is whether this variety in substrates also leads to a corresponding diversity in the microbiota. The papers in this Topic do not allow a profound meta-analysis necessary to formulate general conclusions. Nevertheless, the presented studies on the endogenous microbiota of BSFL during rearing cover a range of substrates, such as cottonseed press cake (Tegtmeier et al.), brewers' spent grain, kitchen food waste, poultry manure and rabbit manure (Tanga et al.), canteen waste and oil separator waste (Klammsteiner et al.), chicken manure and chicken waste (Shumo et al.). They all used (at least) amplicon sequencing of the whole microbiota to obtain the relative abundances of genera present.

In addition to the microbiota as it occurs during the routine rearing of insects, a contribution to the Topic by Yang et al. described the substantial impact of interventions during rearing, i.e. starvation, on the composition as well as the predicted metabolic functions of the microbiota. Studying the reaction of the microbiota on such interventions can help to optimize rearing practices toward maximal biomass yield or conversion efficiency. Whereas Yang et al. used the well-defined and research-oriented Gainesville diet for their starvation study, the effect of such interference when using industrial waste streams is a subject for future research.

Another dimension in the research on the insect microbiome during rearing relates to the addition and monitoring of a particular species of microorganisms, either an unwanted one or a potentially beneficial one. To monitor food pathogens during rearing in order to evaluate microbiological safety, they are typically inoculated under controlled conditions and then kept track of using selective media. In this way, Lopes et al. inoculated *Salmonella* and *E. coli* during fish waste processing by BSFL. They found these bacteria to be reduced by the larvae, yet the reduction was dependent on the feeding regime. Jensen et al. exposed mealworm larvae (*Tenebrio molitor*) to a flour-based substrate contaminated with *Salmonellla* at several inoculation levels. Except at the lowest contamination level, *Salmonella* remained present in the larvae throughout the 14-days rearing period. This indicated that using substrates free of *Salmonella* is a key measure in the production of microbiologically safe mealworms. When investigating the fate of food pathogens during insect rearing, the implications on the microbiological end product safety are the main concern, and not the impact of the food pathogens on insect growth performance. This is in contrast with studies investigating the effect of the addition of potential probiotic bacteria during rearing, such as that of Kooienga et al. They tested the effect of *Arthrobacter, Bifidobacterium breve* and *Rhodococcus rhodochrous* cultures on growth performance, conversion efficiency, and microbial population structure and function of BSFL grown on the Gainesville diet at laboratory and industrial scales. At both scales, the performance of the larvae was improved, but the authors mentioned that care should be taken towards the appropriate bacterial supplement and more trials are needed for proper confirmation of the probiotic effect.

Reared insects reside in an environment with a high microbial load and hence they tend to contain a high number and a wide diversity of microorganisms. Typical processes to transform the insects into a stable end product suitable for a certain application include a large reduction in numbers and/or a change in microbial composition. This is illustrated in this Topic by Fröhling et al. in the flour production process from crickets (*Acheta domesticus*), consisting of freezing, thawing, washing, heating, drying, and pulverization steps. Evidently, microbial numbers were highly reduced after this processing chain, but a small microbiota was still present and grew on selective media for a range of food pathogens, indicating that some microbiological risk possibly remained. Borremans et al. studied the stabilization of blanched and pulverized mealworms. Fermentation of the paste appeared to be a better approach to control the residual microbiota in the paste than applying traditional meat preservatives. These two Topic contributions show that the microbial dynamics during insect processing are highly dependent on the insect species in combination with the processing technologies (and the concomitant process parameters) applied. Generalizing and extrapolating conclusions from one insect species to another and from one processing technique to another is difficult or even impossible.

Finally, the topic also includes work on the microbiology of insect-based end products. Obviously, the insect biomass is the main end product, but the residue (feces, unconsumed substrate, and/or exuviae) is another end product. Mao et al. explored for the first time the microbiota of tea prepared from the feces of meal moths (*Pyralis farinalis*) reared on a specific plant-based diet. The microorganisms mainly originated from the insects and not from the plants they were reared on, and they appeared to survive the roasting and the brewing of the tea. Residues (frass) from BSFL rearing are increasingly being investigated and applied as soil improvers. Some microorganisms are thought to play a role here, but research so far is mainly directed to unraveling the composition of the microbiota of the frass. As BSFL can be grown on a wide variety of substrates, the microbiological quality of BSFL frass can also be different. In this Topic, Gold et al. described the microbiota of frass of BSFL produced using canteen food waste and household food waste. Some typical members of the BSFL core microbiome, such as *Providencia, Dysgonomonas, Morganella*, and *Proteus*, appeared also to be abundant in the frass. Research on the microbiology of insect frass is only in its infancy now, but knowledge about the composition and role of microorganisms in frass may contribute to upcycling frass as a side stream of insect production, improving its economic value and giving it a significant place in a circular economy.

## Author Contributions

All authors listed have made a substantial, direct and intellectual contribution to the work, and approved it for publication.

## Conflict of Interest

The authors declare that the research was conducted in the absence of any commercial or financial relationships that could be construed as a potential conflict of interest.

## Publisher's Note

All claims expressed in this article are solely those of the authors and do not necessarily represent those of their affiliated organizations, or those of the publisher, the editors and the reviewers. Any product that may be evaluated in this article, or claim that may be made by its manufacturer, is not guaranteed or endorsed by the publisher.

